# Differential Experiences of Mental Health among Trans/Gender Diverse Adults in Michigan

**DOI:** 10.3390/ijerph17186805

**Published:** 2020-09-18

**Authors:** Shanna K. Kattari, Leonardo Kattari, Ian Johnson, Ashley Lacombe-Duncan, Brayden A. Misiolek

**Affiliations:** 1 School of Social Work, University of Michigan, Ann Arbor, MI 48109, USA; lacombed@umich.edu; 2 Department of Women’s and Gender Studies, University of Michigan, Ann Arbor, MI 48109, USA; 3 School of Social Work, Michigan State University, East Lansing, MI 48824, USA; kattaril@msu.edu; 4 School of Social Work, University of Washington, Seattle, WA 98105, USA; ianmj@uw.edu; 5 Transcend the Binary, Detroit, MI 48220, USA; brayden@transcendthebinary.org

**Keywords:** depression, anxiety, suicidality, non-suicidal self-injury, transgender, mental health, gender diverse, nonbinary, gender identity

## Abstract

Transgender and gender diverse individuals experience high rates of health disparities, as compared with their cisgender (non-transgender) counterparts. One area in which these disparities is most grave is that of mental health, with some studies indicating transgender and gender diverse individuals as having a 40% rate of lifetime suicide attempts and similarly high rates of depression, anxiety, and suicidal ideation. These rates vary further within this population, with differential rates seen across sociodemographic factors, including race/ethnicity, gender, sexual orientation, disability status, education level, and income. This study explores mental health experiences across different social identities, using data from the 2018 Michigan Trans Health Survey (*N* = 659), a community-based participatory action research project collected in Michigan, United States, analyzed using chi-square tests of independence and logistic regressions. Findings indicate incredibly high rates of mental health concerns; 72.2% had been diagnosed with depression in their lifetime and 73.0% had been diagnosed with anxiety in their lifetime. In the past year, 49.9% had had non-suicidal self-injury (NSSI) thoughts, 45.4% had suicidal thoughts, 26.3% engaged in NSSI, and 7.7% had attempted suicide. Bivariate regressions showed some nuanced experiences of rates of mental health diagnoses and experiences, such as greater odds of experiencing all mental health disparities among those with disabilities, and differential odds across gender in regard to ever having a depression diagnosis, non-suicidal self-injury thoughts and engaging in non-suicidal self-injury behavior. This indicates a need for social workers, counselors, therapists, and other human services professionals to act more intentionally and with an intersectional lens when it comes to exploring the mental health of transgender and gender diverse persons.

## 1. Introduction

### 1.1. Transgender and Gender Diverse Identities

Transgender (trans) and gender diverse (TGD) people are a diverse group of individuals whose gender identity and/or expression do not align with societal expectations associated with the sex they were labelled at birth [1,2]. TGD individuals may include those who identify as transmasculine/trans men/men, transfeminine/trans women/women, in addition to individuals whose genders are more fluid and/or may not fit within a masculine/feminine binary (e.g., nonbinary, genderqueer, agender). Given that these definitions may vary across individuals, it is logical that prevalence rates vary source to source. However, recent estimates suggest that between 0.4% and 0.6% of adults in the U.S. have a TGD identity [1,2]. A plethora of research has identified mental health disparities among TGD individuals, due in part to stigma and discrimination faced by TGD communities. Specifically, studies have identified disparate rates of depression, anxiety, and suicidality compared to cisgender (cis) (non-transgender) persons, those whose gender identity and sex labelled at birth are congruent and who do not experience such stigma. However, scant literature has explored how depression, anxiety, and suicidality differ within TGD communities across sociodemographic characteristics (e.g., race/ethnicity, gender, sexual orientation, disability status). This paper seeks to fill this gap, in order to inform future population-specific interventions at micro, mezzo, and macro levels.

### 1.2. Mental Health among Adults in General in the United States

Depression can include mood shifts away from happiness, diminished interest in life activities, changes in appetite or weight, an observable slowing down of thoughts and movements, fatigue, feelings of worthlessness and guilt, loss of concentration, and thoughts of suicide [3]. Clinical depression is a collection of these symptoms sustained for at least a two-week period to an extent that causes functional disruption and cannot be associated with organic causes or a major life adjustment, including bereavement [3]. Symptoms of depressive disorders can carry an incredible social and emotional burden for individuals, families, and communities, and causes an estimated national economic burden of upwards of USD 210.5 billion [4]. Over 20% of adults in the United States report experiencing clinical depression in their lifetime [5]. Epidemiological studies have indicated demographic disparities in depression prevalence among women [5,6] and people experiencing poverty [5]. In the adult population at large, levels of depression prevalence have been found to be higher among white people [5], though critiques have arisen from studies on measurement tools adequately capturing differential cultural expressions [7], histories of community surveillance surrounding mental illness [8] and epidemiological studies with subgroups, such as those over 65 years of age [9]. Regardless of discrepancies, being of a race other than White is associated with facing more barriers to mental health treatment [10] and depressive symptoms having a larger functional impact on daily life [5]. Experiencing poverty, being 18–29 years of age, and not being male have also been found to negatively affect access to mental health treatment [11,12]. In general, levels of treatment across all U.S. populations are seen as suboptimal [13], and can be attributed to lack of access as well as attitudinal and behavioral factors [14,15].

Innumerable factors across the life course have been attributed to depression in the United States adult population, ranging in scale from epigenetic factors [16,17] personality [18] to cultural [19] and geographic [20]. Notably, stigma has been examined as a factor for depression [21]. Numerous studies have indicated stigma as a cause of or an exacerbating variable in depression, including sexual orientation [22] weight [23], HIV/AIDS [24,25], race and ethnicity [26,27,28,29], disability [30], and citizenship status [31]. Anticipated stigma surrounding mental health can be a cause for delaying, avoiding or disengaging in treatment [32].

Anxiety can be a normal and functional response to cues in the environment, and anxiety disorders refer to when these responses are overactive, misplaced, and/or interfering with life tasks [33]. Symptoms of anxiety may include physiological symptoms (e.g., muscle tension, heart palpitations, sweating, respiratory constriction, digestive pain, insomnia), psychological symptoms (e.g., excessive worry, inattentiveness, perseveration on source of anxiety, thoughts of death or dying), and changes in behavior (e.g., avoidance, hypervigilance, changes in relational attachment) [3]. Anxiety disorders are the most prevalent cluster of mental disorders; estimates of lifetime prevalence range between 8–30% of the global adult population [34]. Anxiety disorders are the sixth-leading global cause of disability [35] and is believed to increase the risk of several health conditions, including chronic pain, metabolic syndromes, cardiovascular problems, and behavioral health conditions, such as smoking, that can lead to medical problems and increase mortality [36,37,38]. The impact of anxiety disorders below the diagnostic threshold on greater health outcomes and disease burden is also significant [39].

Anxiety in adulthood is associated with adverse life events both in childhood and throughout the adult life course [40]. Factors, such as personality traits, parental affect, genetic predisposition, comorbidity with physical illness and other mental health conditions [41]. Similarly with depression, anxiety rates are reportedly lower in Hispanic/Latinx and African-American populations, but with debate; psychological processes, including the awareness of racism, mental health stigma, and prevalence of health inequalities, are believed to cause differences in anxiety expression in ways that prevent diagnosis [42]. Hofmann, Asnaani, and Hinton [43] also observe significant cultural differences in how anxiety manifests and is expressed in collectivist cultures. The impact of anxiety disorders on population health is also believed to be more pronounced on marginalized racial and ethnic groups [44], and sexual minorities [45].

In the general population, the lifetime prevalence of suicidal ideation is approximately 9%, with 3% leading to suicidal planning. Sociocultural context, access to lethal means, access to mental health services, genetics, early life adversity, impulse aggression and cognitive factors, life events, psychopathology, behavioral disinhibition, and social network support can all contribute to the presence of suicidal thought and the extent to which self-harm is carried out [46]. Alcohol and substance abuse have also been identified as a behavioral indicator of increased risk for both suicidal ideation and suicidal attempt, though suspected variation exists between suicide risk and type of drug, as well as method of intake [47].

Suicidality is associated with a variety of mood states. Anxiety and depression are both associated with suicidal attempts [48,49]. Panic disorders and post-traumatic stress disorder are each associated with higher likelihood of suicide attempts, and comorbidly, significantly associated with an even higher likelihood of suicide attempts. Those who experience psychosis or cyclothymic mood disorders are also more likely to experience suicidal ideation or attempts [50,51]. Hopelessness is a significant indicator of the likelihood of suicidal ideation [49]. Both the internal experience of anger and the outward expression of anger and aggression can be predictive of suicidal thought and attempts [52]. Kleiman et al. [53] also note that suicidal ideation and its associated mood states, such as hopelessness, burdensomeness, and loneliness, can vary greatly on a daily or even hourly basis. Research has also suggested that, independent of existing mental disorder, suicidal events are often predicated by adverse life events, such as interpersonal conflict, the death of a loved one, arrest or police intervention, or loss of employment [54]. Many risks associated with suicide include populations facing systemic injustice surrounding race, employment status, education, and demographic, though the significance of such identities varies with age [55]. While protective factors for suicidality have historically been under-researched, there are indications that there are environmental and contextual factors, such as religiosity [56] and social support [57].

### 1.3. Mental Health among TGD Adults in the United States

Studies have identified many negative mental health consequences of stigmatization and of trans and non-binary persons, including nearly ubiquitous depression, high rates of anxiety, and epidemic-level suicidality. As many of the factors associated with depression, anxiety, and suicidality are overlapping, we first discuss the prevalence of each of these three conditions, followed by associated factors.

High rates of depression have been consistently identified in TGD populations [58,59,60,61]. For example, in a U.S. based study, 44.1% of 1093 trans people had clinical depression [58]. Another study, focused on trans women/girls aged 14 to 25, reported that over two-thirds (68.3%) of the sample experienced a major depressive episode in the past year [62]. Studies have also shown higher rates of depression among trans youth compared to their cis peers [59,63]. In illustration, Reisner et al. [63] identified a more than double probability of having a physician-endorsed diagnosis of depression among trans youth compared with matched cis controls (50.6% vs. 20.6%).

Similar to depression, high rates of anxiety have also been described among TGD populations [58,64,65,66]. A recent systematic review was published exploring the prevalence of anxiety symptoms and disorders in the trans population, drawing on data from 17 cross-sectional and 8 longitudinal studies [66]. The prevalence of anxiety disorders ranged from 17% to 68%, with the most commonly reported anxiety disorders including specific phobias, social phobias, panic disorders, and obsessive-compulsive disorders [66]. Generalized anxiety disorder rates are also high among TGD persons [65]. For example, a study of 109 adolescent TGD people showed that 48% met diagnostic criteria for generalized anxiety disorder [65]. Moreover, studies have shown higher rates of anxiety among trans people compared to cis people [64]. Among a sample of 592 trans people, 68.8% had possible anxiety disorders, compared to 32.8% of matched cisgender controls [64].

Data from the 2015 population-based National Transgender Health Survey (NTHS) (*n* = 27,715), showed that 40% of respondents had attempted suicide in their lifetime and 7% had attempted suicide in the past year [67]. More than two-thirds (71%) of respondents who had attempted suicide in the past year had done so more than once in their lifetime, with almost half (46%) reporting having attempted suicide three or more times. These statistics show the chronicity with which suicide pervades the lives of TGD people. With respect to ideation, the proportions are even starker, such that 82% of respondents reported serious thoughts about suicide at some point in their life and 48% of respondents reported having serious thoughts about suicide in the past year [67]. Drawing on nationally-representative US data, Perez-Brumer et al. [68] found that trans youth had almost three times the odds of reporting past-year suicidal ideation compared to cis youth. Newcomb et al. [69] also examined differences between gender identities in suicidality. The authors found that nonbinary youths assigned male at birth reported the highest rates of suicidal ideation (50.0%), compared to nonbinary youths assigned female at birth (31.7%), as well as trans men (35.7%), or trans women (13.8%) [69].

### 1.4. Factors Associated with Mental Health Outcomes among Trans and Gender Diverse Adults

High rates of negative mental health outcomes among TGD people are associated with a myriad of social and structural factors that work to impede or promote mental health. The most robust evidence lies in our understanding of how stigma, social exclusion and violence contribute negatively to mental health outcomes among trans people [61,63,70,71,72]. For example, Nuttbrock et al. [73] reported a strong association between psychological and physical gender-related abuse and major depression among 571 trans women. Moreover, they found that the effects of both types of abuse were greatest during adolescence [73]. Findings from the USTS also showed higher rates of attempted suicide among those who reported their families as unsupportive and those had experienced physical or sexual violence [67]. Transphobia also intersects with systemic racism, contributing to higher rates of depression, anxiety, and suicidality among trans and non-binary people of color [67,74]. Trans and non-binary people with disabilities are also at elevated risk of suicidality [67].

Alternately, social support and resilience are associated with positive mental health outcomes [58,62]. Moreover, access to gender affirmation, including medical (e.g., access to hormones), social (e.g., proper use of one’s name and pronouns), and legal (e.g., ability to change one’s identification) means of affirming one’s gender identity, can promote mental health among trans people [64,75,76]. For example, a study by Kattari et al. [76] found that 37.8% of trans people who reported having a trans-inclusive provider reported current depression, compared to 53.7% of those who did not report having a trans-inclusive provider. These findings also held for anxiety and suicidal thoughts in the past year [76]. TGD people living in rural communities report additional challenges, such as limited access to proximally-located and affirming providers [77]; not surprisingly, studies have shown that rural trans people experience higher rates of depression and suicidality compared to non-rural trans people [78].

### 1.5. Research Question

Given the limited research on TGD youth and their overall experiences of sexual assault and dating violence, we pose the question: (1) How do TGD adults’ experiences of mental health challenges vary across gender identity, race, age, sexual orientation, and geography?

## 2. Materials and Methods

### 2.1. Methods and Procedures

The Michigan Trans Health Survey (MTHS) was conducted in 2018 as a partnership between the first author, and the TGD focused community organization Transcend the Binary (TtB). Survey items related to identities, mental health, physical health, access to resources, intimate partner violence, etc. were created January–March 2018 by the investigators, TGD individuals that participated a focus group led by TtB, and other TGD-focused health researchers who are part of the Transgender Research Group at the University of Michigan. After all survey items were co-created, members of TtB looked over the final draft of the survey to offer input on language, ordering of items, and suggestions on the text to include throughout for framing. Following this, ten TGD individuals from outside the state (so as not to preclude them from taking the survey once it opened) conducted cognitive interviews regarding flow, length of time to take the survey, language, and cultural responsiveness. One additional cognitive interview was run with a blind (cis) individual in order to ensure that the survey was screen reader compatible for full accessibility. The average amount of time reported to take the survey was between 25 and 30 min, with some participants completing it in less than 15 min, and some taking closer to 60 min. The survey was determined to be exempt via [REDACTED] Institutional Review Board. Participants were given USD 10 Mastercard gift cards on completion of the survey.

In order to participate in the survey, participants had to be 18 years of age or older, live in the state of Michigan, and identify as “transgender, trans, non-binary, genderqueer, agender, genderfluid, two-spirit, transsexual, or another non-cisgender identity.” They also had to consent in order to take the survey. Participants were recruited May through September 2018, online and in person, throughout the state of Michigan to take the 2018 Michigan Trans Health Survey online. Recruitment occurred via Facebook, Twitter, websites, email, and listservs to disseminate the survey online (both organically and with paid ads), and study team members handed out palm card and business size recruitment cards at various transgender events, Pride events, community organizations, and health provider’s offices throughout the state. Snowball sampling, where participants shared this survey with their own networks after taking it, seemed to be common, in addition to the general convenience sampling that was the primary recruitment effort. Several community organizations also offered paper copies of the survey; no paper copies were returned.

Initially, the data were cleaned for exclusionary/ineligibility criteria, duplicate responses, and illogical responses. Additionally, the qualitative responses to sociodemographic categories (gender, sexual orientation, race/ethnicity, and religious/spiritual identities) were coded into categorical variables for use in quantitative analysis.

### 2.2. Measures

The predictor variables were collected on the MTHS as sociodemographic information that had a large range of response selections that was cleaned and coded by the MTHS team and then re-coded for this analysis. Gender identity was condensed from eight values into four by combining female/woman and trans feminine into one value for feminine genders, male/man and trans masculine into one value for masculine genders, nonbinary as its own value, and trans/transgender, multiple gender and additional genders into one value capturing identities that vary from more common language use. Sexual orientation was condensed from ten values into six values by combining gay and lesbian into one value that captures single gender attraction identities; combining bisexual, omnisexual, and pansexual into one value that captures multiple gender attraction sexualities; straight/heterosexual as its’ own value; asexual/demisexual as its’ own value; combining multiple orientations, additional orientations, and homosexual into one value to capture identities that vary from more common language usage. Racial identity was re-coded from nine values to five values to include Black/African American, Latinx/Chicanx/Hispanic, White, Biracial/Multiracial, and an additional racial identity value that combined Asian, Asian American, Pacific Islander, Native, Indigenous, American Indian, Middle Eastern, Romani and additional races, as responses within these racial identities were too small to keep separate due to confidentiality concerns and statistical limitations. The geographic locality variable was re-coded from five values to a bivariate value by combining urban and suburban into one value to capture more densely populated areas and combining small city, rural and frontier into one value to capture less densely populated areas. Age as a continuous variable and a bivariate disability variable (do you have one or more disabilities? yes/no) were also used as predictor variables.

The six mental health outcome variables were re-coded into bivariate variables. The depression diagnosis variable combines “yes, I have been diagnosed in the past twelve months” and “no, but have been told this before” to create “ever been told they have been depressed in their lifetime” and combined values for “No” and “No—I did not receive any health care in the past 12 months” to create a never been told value. A similar re-coding was done for an anxiety diagnosis, to create a bivariate variable of “ever been told to have anxiety in their lifetime” yes/no variable. The four other variables asked respondents to consider their mental health behaviors in the past twelve months, including having thoughts about non-suicidal self-injury (NSSI), having thoughts about suicide, engaging in NSSI or attempting suicide. The values for each of these variables were condensed from three values (Yes, No, No - but have in the past) to a bivariate yes or no in the past twelve months variable.

### 2.3. Data Analysis

Data were assessed for missingness, and all of the outcome variables had less than 10% missing data. Chi-square tests of independence were conducted to assess the relationship between the predicator variables (gender identity, sexual orientation, race, geographic region, disability, and age) and the outcome variables (depression diagnosis in lifetime, anxiety diagnosis in lifetime, thoughts about NSSI in the past twelve months, thoughts about suicide in the past twelve months, engaging in NSSI in the past twelve months, and suicide attempt in the past twelve months). The predictor variables that showed significance with the outcome variables were then used in a bivariate logistic regression. This included gender identity, sexual orientation, disability and age. The predictor variables of race and geographic region were dropped from the regression analysis as the chi-square tests did not indicate significance among any of the outcome variables. Suicide attempts was also dropped from the regression analysis because it demonstrated a relationship with only disabled respondents.

## 3. Results

### 3.1. Sample Characteristics

Descriptive characteristics of sample (Table 1) are shown for all respondents of the MTHS (*n* = 659). The mean age of the sample was 28.58 (SD: 0.41). Thirty percent of the sample identified as a man/transman/transmasculine, 19.4% as a woman/transwoman/transfeminine, a quarter as non-binary, and 21.7% as an additional gender. Over a quarter (27.6%) of respondents identified as bisexual, omnisexual or pansexual, 18.5% identified as queer, 17.5% as gay or lesbian, 14.4% as additional orientations, 12% as straight and 5.6% as asexual or demisexual. A large majority of the sample identified as White (76.5%) and lived in an urban/suburban area (69%). Almost half (43.2%) indicated that they have a disability.

### 3.2. Mental Health Outcomes

Among the outcome variables, 72.2% had been diagnosed with depression in their lifetime and 73.0% had been diagnosed with anxiety in their lifetime. About half (49.9%) had had NSSI thoughts in the last 12 months, 45.4% suicidal thoughts in the last twelve months, a quarter (26.3%) engaged in NSSI in the last twelve months, and 7.7% attempted suicide in the last twelve months. See Table 2 for more all results.

### 3.3. Differences in Mental Health Outcomes by Sociodemographic Characteristics

The bivariate regression (Table 3) shows the five models holding all predictor variables constant. Across all five models—ever been diagnosed with depression (OR = 2.03, *p* < 0.001), ever been diagnosed with anxiety (OR = 3.09, *p* < 0.001), NSSI thoughts (OR = 1.56, *p* < 0.05), suicidal thoughts (OR = 2.14, *p* < 0.001), and engaging in NSSI (OR = 2.03, *p* < 0.001)—those with disabilities had statistically significant greater odds at experiencing mental health disparities. Among those who identify as nonbinary, had greater odds of ever being diagnosed with depression (OR = 1.93, *p* < 0.01), having NSSI thoughts (OR = 1.74, *p* < 0.05) and engaging in NSSI (OR = 0.98, *p* < 0.01). Additionally, those that identify their gender identity with additional genders had less likely odds of experiencing NSSI thoughts (OR = 0.56, *p* < 0.05) compared to transmasculine individuals. Age, Age2, and sexual orientation were not significant for any of the outcome variables. Given that the chi square tests of independences indicated no significant differences across race/ethnicity or place of residence (urban/suburban compared with small town/rural), these variables were dropped from the regression analysis.

## 4. Discussion

This study sought to identify differences in mental health experiences across diverse social identities held by TGD people. Much research published to-date has treated TGD communities as a monolith, examining mental health differences between TGD and cis people, as opposed to exploring differences within the TGD community. Understanding these differences can inform targeted support for TGD people with specific identities. Specifically, we identified mental health disparities among TGD people with disabilities, straight/heterosexual TGD people, and nonbinary TGD people.

Contrary to other studies [74,78] our findings did not show significant differences across race/ethnicity or place of residence (urban/suburban compared with small town/rural), these variables were dropped from the regression analysis. It is possible that this lack of significance had to do with the sample size and need to combine certain responses in order to have enough power for the bivariate analyses. One previous study, somewhat limited in how it operationalized gender, found that mental health disparities for transgender adults have been found to be magnified in rural populations [74]. Smith et al. [79] found that conservative community beliefs, restrictive local policies, and geographic barriers to accessing trans-affirming care may contribute to mental health disparities, while interpersonal factors, such as a strong support system, may serve as a more influential protective factor for rural TGD than TGD people in urban areas. Future studies, particularly those with larger sample sizes, should conduct similar analyses to assess whether this lack of findings was due to sample size, or is indeed representative of this population.

### 4.1. Implications

What is of particular interest about these findings is that, while on their own, many of the sociodemographic identities were significant when run in bivariate analysis, but when placed into a logistic regression, many of this significance disappeared. Gender identity was significant regarding having ever been diagnosed with depression, NSSI thoughts, and NSSI attempts, with nonbinary individuals experiencing almost two times the likelihood of negative mental health outcomes than their transmasculine peers. Similarly, sexual orientation was significant for all of the relevant outcome variables in the chi square test of independence, but no significance was found in the regressions. The only variable that was significant across all of the outcome variables was disability status, with disabled participants being more than five times as likely to have been diagnosed with depression and three times more likely to have been given an anxiety diagnosis, compared to their non-disabled peers. While this may be because many of those responding as having a disability identity included mental health concerns their disability, or as one of their disabilities, it is also worth noting that disabled individuals are also one and a half times more likely to have had NSSI thoughts, more than twice as likely to attempt non-suicidal self-injury in the past year, and more than twice as likely to have had suicidal thoughts as their non-disabled peers, which indicates a deep need to better understand the unique experiences of TGD individuals, from a mental health perspective, and in general. There is little previous research on this intersection, but what does exist indicates that disabled TGD individuals are more likely to experience discrimination when accessing mental health services, making this particularly fraught [80].

Given that almost three quarters of the sample had ever received a diagnosis of depression (72.2%) or anxiety (73.0%), and almost half had experienced thoughts of non-suicidal self-injury (49.9%) or suicide (45.5%) in the past year, it is clear than trans affirming support for mental health concerns is of the utmost importance. It is interesting that there are few sociodemographic characteristics related to the likelihood of these experiences, indicating that many of these mental health experiences are more universal experiences for this population, rather than dependent on other identities and experiences. It must be noted that TGD do not have high rates of mental health concerns simply because of the TGD identities; rather, it is the experiences of transphobia, discrimination, harassment, and victimization, along with the stigma societally associated with these identities, that results in these higher rates [81]. Ergo, practitioners must be especially careful to not simply treat TGD patients as having mental health concerns due to their gender, nor to pretend as if the world is inherently supportive if only they can just accept themselves, but rather, to work at the micro, mezzo, and macro levels to help create a more affirming society that offers TGD individuals a space to thrive, an addition to treating the mental health concerns specifically. More awareness should also be given to disabled TGD individuals, to ensure that the challenges of ableism (the devaluing of disability) faced by their patients are also engaged to ensure they are also as supported as possible.

### 4.2. Limitations

As with all cross-sectional data, these findings represent one snapshot in time, rather than a deeper understanding of how various sociodemographic characteristics are related to various mental health outcomes. Additionally, while disability as a category was significant across all outcome measures, disability was used as one larger monolith in this study and could include existing mental health concerns. Future research, using larger sample sizes, should assess whether different types of disabilities have differential likelihoods of increased risk for different mental health outcomes. With a sample size of 659, this required several robust sociodemographic categories to be collapsed together for the purpose of analysis. It would be useful for larger surveys to have more diverse response choices for identity variables (such as race, sexual orientation, gender, etc.) so that these categories would not need to be collapsed for analysis. Finally, while this sample reflected the racial breakdown of the state of Michigan, it still resulted in a sample that was 76.5% White. There is a need for more diverse samples of TGD individuals to better understand the unique experiences of Black, Indigenous and other TGD people of color (BIPOC), and future research should work to intentionally recruit more BIPOC individuals with a goal of oversampling for more nuanced analyses.

## 5. Conclusions

Members of the TGD population experience incredibly high rates of mental health concerns, much of which may be due to navigating a world of stigma, discrimination, and victimization. While, when looked at separately, several sociodemographic variables were significantly related to mental health outcomes, upon being regressed, it appears that disability status universally increases a likelihood of these five mental health outcomes, while gender (nonbinary) is significantly related to ever having a depression diagnosis, NSSI thoughts, and engaging in NSSI compared to transmasculine TGD individuals. All in all, practitioners working with members of this population need to be aware of the universal issues of mental health concerns in this population, paying special attention to their disabled clients and gender identity, and work across policy, interpersonal practice, and systems to support their clients’ mental health.

## Figures and Tables

**Table 1:** Demographic Breakdown (*n* = 659).

**Variable**	***n***	**%**
*Predictors*		
**Gender Identity**		
Woman/Transwoman/Transfeminine	128	19.4
Man/Transman/Transmasculine	199	30.2
Nonbinary/GQ/AG/GF	170	25.8
Additional Genders	143	21.7
Missing	19	2.9
**Sexual Orientation**		
Gay/Lesbian	117	17.8
Bisexual/Pan/Omni	182	27.6
Queer	122	18.5
Straight/Heterosexual	79	12
Asexual/Demisexual	37	5.6
Additional Sexual Orientations	95	14.4
Missing	27	4.1
**Race**		
Black/African American	42	6.4
Latinx/Chicanx/Hispanic	25	3.8
White	504	76.5
Multiracial/Biracial	36	5.5
Additional Races	23	3.5
Missing	29	4.4
**Residence**		
Urban/Suburban	455	69.0
Small City/Rural/Frontier	204	31.0
**Age (Mean/Standard Deviation)**	28.58	0.41
**Disability**		
Yes	285	43.2
No	374	56.8
*Outcomes*		
**Ever Diagnosed with Depression**		
Yes	476	72.2
No	128	19.4
Missing	55	8.3
**Ever Diagnosed with Anxiety**		
Yes	481	73.0
No	123	18.7
Missing	55	8.3
**NSSI Thoughts in Last 12 Months**		
Yes	329	49.9
No	272	41.3
Missing	58	8.8
**Suicidal Thoughts in Last 12 Months**		
Yes	299	45.4
No	302	45.8
Missing	58	8.8
**NSSI in Last 12 Months**		
Yes	173	26.3
No	431	65.4
Missing	55	8.3
**Suicide Attempt in Last 12 Months**		
Yes	51	7.7
No	554	84.1
Missing	54	8.2

**Table 2:** Chi Square Tests of Independence of Demographics by Mental Health Outcomes.

Variable	Ever Diagnosed Depression (*n* = 604)			Ever Diagnoses Anxiety (*n* = 604)			NSSI Thoughts (*n* = 601)			Suicidal Thoughts (*n* = 601)			NSSI (*n* = 604)			Suicide Attempt (*n* = 605)		
*Predictor*	*N*	%	*p*	*N*	%	*p*	*N*	%	*p*	*N*	%	*p*	*N*	%	*p*	*N*	%	*p*
**Gender Identity**			*			***			***			*			***			
Woman/Transwoman/Transfeminine	83	68.6		81	66.9		50	41.3		48	39.7		23	19.0		8	6.5	
Man/Transman/Transmasculine	138	9.8		134	7.5		85	49.1		87	50.6		41	23.7		14	8.1	
Nonbinary/GQ/AG/GF	134	83.8		144	90.0		108	67.9		93	58.1		68	42.5		17	10.6	
Additional Genders	113	85.6		113	85.6		81	61.8		65	50.0		36	27.3		9	6.8	
**Sexual Orientation**			*			*			***			*			**			
Gay/Lesbian	90	82.6		87	18.6		65	60.2		49	45.4		28	25.7		4	3.7	
Bisexual/Pan/Omni	133	8.7		136	80.5		92	54.8		91	53.8		54	32.0		20	11.8	
Queer	89	80.2		97	87.4		73	65.8		65	58.6		40	36.0		9	8.1	
Straight/Heterosexual	44	63.8		47	68.1		23	33.3		22	32.4		8	11.6		4	5.8	
Asexual/Demisexual	28	85.3		30	88.2		21	65.8		19	55.9		13	38.2		4	11.8	
Additional Sexual Orientations	74	86.0		71	82.6		49	57.0		44	51.8		24	27.9		6	7.0	
**Race**																		
Black/African American	28	71.8		28	71.8		16	41.0		15	38.5		10	25.6		5	12.8	
Latinx/Chicanx/Hispanic	17	70.8		18	75.0		10	41.7		12	50.0		7	29.2		3	12.5	
White	370	79.9		378	81.6		264	57.1		227	49.3		131	28.3		37	8.0	
Multiracial/Biracial	25	80.6		24	77.4		16	53.3		18	58.1		9	29.0		1	3.2	
Additional Races	20	95.2		18	85.7		15	71.4		15	71.4		10	47.6		2	9.5	
**Residence**																		
Urban/Suburban	330	79.1		334	80.1		217	52.4		202	48.8		118	28.3		41	9.8	
Rural/Small City/Frontier	146	78.1		147	78.6		112	59.9		97	51.9		55	29.4		10	5.3	
**Disability**			***			***			**			***			***			***
Yes	237	91.2		233	89.6		158	61.5		160	61.8		100	38.5		35	13.4	
No	239	69.5		248	72.1		171	49.7		139	40.6		73	21.2		16	4.7	

Note: * *p* < 0.05, ** *p* < 0.01, *** *p* < 0.001.

**Table 3:** Binary Logistic Regressions of Mental Health Outcomes.

Variables	Ever Diagnosed Depression (*n* = 578)		Ever Diagnosed Anxiety (*n* = 578)		NSSI Thoughts (*n* = 576)		Suicidal Thoughts (*n* = 575)		NSSI Attempt (*n* = 578)	
	OR	95% CI	OR	95% CI	OR	95% CI	OR	95% CI	OR	95% CI
**Age**	1.11	[0.99, 1.24]	1.03	[0.92, 1.15]	1.08	[0.98, 1.20]	1.04	[0.95, 1.15]	1.11	[0.99, 1.24]
**Age ^2^**	1.00	[0.999, 1.00]	1.00	[0.999, 1.00]	1.00	[0.998, 1.00]	1.00	[0.999, 1.00]	1.00	[0.998, 1.00]
**Gender Identity** (*Transmasculine)*										
Transfeminine	0.98	[0.52, 1.84]	1.20	[0.66, 2.21]	0.95	[0.56, 1.61]	1.22	[.0.72, 2.07]	0.98	[0.52, 1.84]
Nonbinary	1.93 **	[1.17, 3.20]	1.93	[0.98, 3.77]	1.74 *	[1.08, 2.81]	1.05	[0.66, 1.68]	1.93 **	[1.17, 3.20]
Additional Genders	0.80	[0.46, 1.39]	.551	[0.28, 1.08]	0.56 *	[0.34, 0.93]	1.00	[0.61, 1.64]	0.80	[0.46, 1.40]
**Sexual Orientation** *(Bisexual/Pan/Omni)*										
Gay/Lesbian	1.09	[0.62, 1.92]	0.84	[0.44, 1.62]	0.67	[0.40, 1.13]	1.19	[.71, 1.97]	1.09	[0.62, 1.91]
Queer	1.15	[067, 1.98]	1.39	[0.67, 2.90]	1.50	[0.88, 2.56]	1.17	[0.70, 1.96]	1.15	[0.67, 1.98]
Straight/Heterosexual	1.84	[078, 4.33]	1.07	[0.53, 2.15]	1.43	[0.75, 2.70]	1.68	[0.89, 3.19]	1.84	[0.78, 4.33]
Asexual/Demisexual	0.69	[0.19, 1.74]	0.50	[0.15, 1.64]	0.69	[0.31, 1.54]	0.86	[0.40, 1.87]	0.69	[0.31, 1.53]
Additional Sexual Orientations	0.91	[0.489, 1.67]	0.58	[0.33, 1.41]	0.78	[0.44, 1.36]	0.89	[0.51, 1.54]	0.91	[0.49, 1.67]
**Disability** *(No)*										
Yes	2.03 ***	[1.38, 2.99]	3.09 ***	[1.85, 5.15]	1.56 *	[1.09, 2.25]	2.14 ***	[1.50, 3.04]	2.03 ***	[1.38, 2.99]

Note: * *p* < 0.05, ** *p* < 0.01, *** *p* < 0.001.
